# Evaluation of the chest computed tomography and hemogram data in patients with COVID-19: the importance of thymus

**DOI:** 10.3906/sag-2007-306

**Published:** 2021-06-28

**Authors:** Vefa ÇAKMAK, Atakan YILMAZ, Tuğba SARI, Pınar ÇAKMAK, Mert ÖZEN, Duygu HEREK, Alten OSKAY

**Affiliations:** 1 Department of Radiology, Faculty of Medicine, Pamukkale University, Denizli Turkey; 2 Department of Emergency Medicine, Faculty of Medicine, Pamukkale University, Denizli Turkey; 3 Department of Infectious Diseases and Clinical Microbiology, Faculty of Medicine, Pamukkale University, Denizli Turkey

**Keywords:** COVID-19, pneumonia, chest, thymus, lymphocyte, platelet

## Abstract

**Background/aim:**

To investigate the relationship between imaging findings and peripheral blood cell counts of COVID-19 patients and the degree of thymus fat involution of these patients.

**Materials and methods:**

Computed tomography (CT) images of 87 patients with COVID-19 positive through RT-PCR testing were evaluated retrospectively by two radiologists. Ground glass densities and other signs of viral pneumonia were recorded, lung involvement was scored quantitatively. The patients thymus fat involution was graded on CT. Neutrophil-lymphocyte ratio (NLR), platelet-lymphocyte ratios (PLR), lymphocyte and platelet counts were calculated. Imaging findings and degrees of thymus fat involution were compared with laboratory data.

**Results:**

Quantitative scoring of lung involvement was calculated at mean 6.63 ± 4.70 (1–23) for observer 1 and mean 6.55 ± 4.65 (1–23) for observer 2 ( *K* = 0.824–1.000). Statistical significance was determined between the increase in age and the increase in scores of lung findings ( *p* = 0.003). Lymphocyte count ( *p* = 0.0001) and PLR ( *p* = 0.001) were significantly lower in patients with severe CT involvement. A statistically significantcorrelation was found between increased thymus fat component and presence of COVID-19 lung involvement in CT ( *r* = 0.461).

**Conclusion:**

The severity of imaging findings for COVID-19 patients significantly correlates with the degree of fat involution in patients’ thymus tissue.

## 1. Introduction

Having first appeared in Wuhan province of China in December 2019, the outbreak of coronavirus disease (COVID-19) has led to the deaths of hundreds of thousands of people worldwide [1]. While the World Health Organization (WHO) declared this situation as a pandemic on 11 March 2020 World Health Organization (2020). Coronavirus disease 2019 (COVID-19) situation report-51 [online]. Website https://www.who.int/docs/default-source/coronaviruse/situation-reports/20200311-sitrep-51-covid-19.pdf?sfvrsn=1ba62e57 [accessed 02 April 2020]., the first COVID-19 cases in Turkey were detected in March 2020 Turkish Government, Ministery of Health (2020). Türkiye’deki Güncel Durum [online]. Website https://covid19.saglik.gov.tr/ [accessed 02 April 2020].. Coronaviruses found in the etiology of the disease are positive-sensitive RNA viruses within the Coronaviridae family. Two other important diseases in the etiology of coronaviruses are severe acute respiratory syndrome (SARS),which broke out in southern China between 2002 and 2003, and the Middle East respiratory syndrome (MERS), which continuedtill 2019 in Saudi Arabia [2]. The clinical findings of COVID-19 are generally reported as high fever, weakness, muscle pain, dry cough and shortness of breath. The reported labresults are varied, and lymphopenia and thrombocytopenia are observed in severe cases [3]. The definitive diagnosis of 2019-novel coronavirus is made by reverse-transcriptase polymerase-chain reaction (RT-PCR) and computed tomography (CT) is known to occupy a key role in the diagnosis of COVID-19. The characteristics of imaging findings and thoracic CT features during the onset and progression of the disease have been defined [4–7] The sensitivity and specificity of thorax CT in the diagnosis of COVID-19 was reported as 97% and 57%, respectively [8].

Some recent research has revealed that neutrophil-lymphocyte (NLR) and platelet-lymphocyte ratio (PLR) between severe and nonsevere cases in COVID-19 can prove effective in the prognosis of the disease [9–11]. Indeed, prognostic factors play a key role in this regard as no effective treatment exists for COVID-19 thus far. 

Thymus is the primary lymphoid organ supporting T lymphocyte development, and normal thymus tissue as well as fat infiltration is readily distinguishable in cross-sectional imaging. As a result of irregularity in the cell-mediated immune system and fatty involution of the thymus, susceptibility to infections increases, and immunity to vaccines decreases [12].

The major aim of the present study is to gain an insight into the relationship between COVID-19 imaging findings and NLR, PLR and peripheral blood cell numbers, in addition to the link between laboraty and imaging findings and thymus tissue of COVID-19 patients.

## 2. Materials and methods

### 2.1. Study group

Thoracic computed tomography (CT) images of the patients presenting to the Emergency Department (ED) of Pamukkale University Hospital with complaints, such as fever, sore throat, dry cough, and shortness of breath were assessed retrospectively. The diagnosis of COVID-19 was confirmed as positive following the RT-PCR test of the nasal and pharyngeal swab samples of these patients taken under the guidelines of WHO. The medical history, examination findings and labresults of our patients were obtained from the hospital medical records. Of all these patients, those with hematological or oncological disease, interstitial lung disease, and diffuse respiratory motion artefacts in their images were excluded from the study. CT images of 87 patients (53 males, 34 females, median age 45, mean 46.31 ± 17.31 years) diagnosed with COVID-19 by RT-PCR test were assessed by two radiologists with board certification.

### 2.2. Computed tomography

CT examinations were performed with a 16-slice helical CT scanner (Brilliance; Philips Medical Systems, Cleveland, OH, USA) and 2 slice helical CT scanners (GE Brivo, Milwaukee, USA). These CT scanners were used only for COVID-19 patients during the pandemic period by taking the necessary disinfection and isolation precautions. Planned on a scenogram image, CT examinations wereperformedwhile the patients were in the supine position and inspiration, with their arms next to their heads. Besides, intravenous contrast media were not used. Thorax CT imaging parameters in 2-slice helical CT scanner were tube voltage 120 kV, tube current 100 mAs, collimation 2 × 1.5 mm, and pitch 0.938, while those in 16-slice helical CT scanner were tube voltage 120 kV, tube current 100 mAs, collimation 16 × 0.75 mm, and pitch 1.063. The CT images of all the patients were evaluated in mediastinum (WW: 350, WL: 50) and parenchyma (WW: –600, WL: 1600) window on the workstation.

### 2.3. Imaging analysis

**Figure 1:**
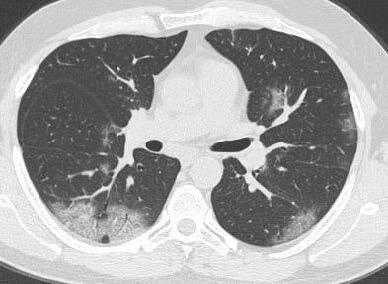

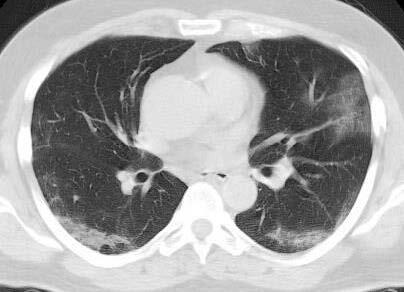

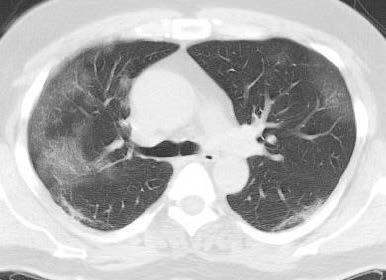

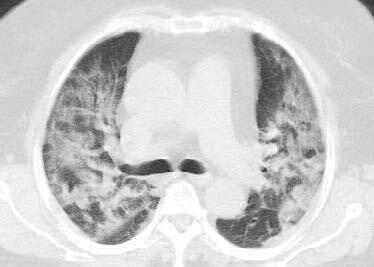
Each lung lobe and lingula (six lung zones) were scored quantitatively (0–24 points). 1 point (1%–25% involvement) for the inferior lobe of the left lung. Ground-glass opacities with reticular pattern with placement of the superior segment of the inferior lobe of the right lung. b. Each lung lobe and lingula (six lung zones) were scored quantitatively (0–24 points). 2 points for left lung superior lobe (25%–50% involvement). c. Each lung lobe and lingula (six lung zones) were scored quantitatively (0–24 points). 3 points forthe superior lobe of the right lung (50%–75% involvement). d. Each lung lobe and lingula (six lung zones) were scored quantitatively (0–24 points). 4 points forthe superior lobe of the right lung (75% or more involvement).

**Figure 2:**
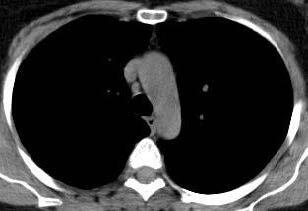

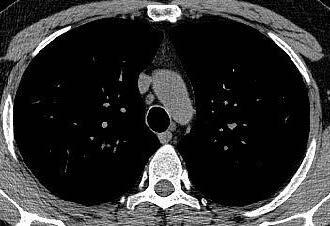

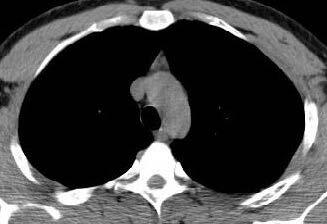

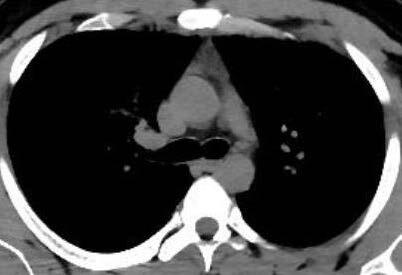
Axial thorax CT images in which the degree of thymus atenuation is assessed. Grade 3. Complete fatty degeneration of the thymus. b. Axial thorax CT images in which the degree of thymus atenuation is assessed. Grade 2. Thymus gland is mostly fatty and reticulonodular densities are observed. c. Axial thorax CT images in which the degree of thymus atenuation is assessed. Grade 1. The thymus is triangular in shape and consists of half solid and half fatty tissue. d. Axial thorax CT images in which the degree of thymus atenuation is assessed. Grade 0. Solid thymus tissue consisting of the majority soft tissue.

The descriptive findings of viral pneumonia described by the Fleischner Society were investigated in the CTs of 87 positive COVID-19 patients between March 15, 2020 and April 15, 2020 [13]. Accordingly, COVID-19 positive patients were classified as with and without lung findings during the admission to the ED. The presence of ground-glass densities and the lobes where they are located were evaluated and recorded for lung involvement. What was probed, except for ground-glass densities, was the presence of consolidation or nodule, crazy paving, halo sign, enlarged vessel sign and air buble sign. Apart from all these, the presence of pleural or pericardial effusion as well as of lymphadenopathy (short axis size greater than 1 cm) and their localizations were assessed in thorax CT images. We quantitatively scored lung involvement of COVID-19 positive patients [14,15]. Accordingly, a total of 6 zones (3 lobes in the right lung, 2 in the left, and lingula) were scored between 0 and 4 points, based on the involvement percentage. As shown in Figures 1a–1d, the scoring scale for lung involvement was devised as such: 0 points = no involvement, 1 point = involvement below 25%, 2 points = 26%–50% involvement, 3 points = 51%–75% involvement, and 4 points = 76%–100% involvement. In the light of this scoring, 8 or more points were considered as severe lung involvement. Thymus was classified in accordance with parenchyma attenuation in the thorax CT of all COVID-19 positive patients [12]. The grading system was formulated as followsas shown in Figure 2a; grade 3 = complete fat infiltration, Figure 2b; grade 2 = larger than 50% fat infiltration of the thymus, Figure 2c; grade 1 = less than 50 % fat infiltration of thymus, half solid thymus tissue, and Figure 2d; grade 0 = complete solid thymus tissue. Then, the degree of attenuation of thymus tissue was compared with our scores on CT for imaging findings of the disease. Moreover, the ground glass placement in the group with lung involvement was compared with lobar involvement, consolidation, crazy paving findings and lymphocyte values.

### 2.4. Laboratory data analysis

Before the onset of treatment administration in the ED for the patients, peripheral blood sampling was performed for complete blood count, c reactive protein, biochemical and coagulation parameters. White blood cell count, neutrophil, lymphocyte, monocyte and platelet count in the complete blood count were recorded for the study. Neutrophil-lymphocyte ratio (NLR), platelet-lymphocyte ratio (PLR) and monocyte-lymphocyte ratio (MLO) were calculated according to the routine peripheral blood sample. These data obtained from peripheral blood sample were compared with CT findings and scores in terms of the presence and severity of lung involvement in COVID-19 positive patients.

### 2.5. Statistical analysis

Data analysis was performed using statistical software (SPSS 21 for Windows, Chicago, IL). The descriptive statistics were expressed as mean ± standard deviation in continuous variables and as % in categorical variables. Chi-square test and Fisher’s exact test were run for categorical variables, while the independent samples t-test was preferred to compare the imaging findings and the degree of thymus fat involution. The Mann–Whitney U test was used to compare nonnormally distributed continious variables. Kolmogorov–Smirnov tests were performed to check normal distribution between the groups for continious variables. Univariate logistic regression models were used to evaluate between variables and lung involvement and adjusted odds ratio (ORs) and 95% confidence intervals (CIs) were calculated. Receiver operating characteristic (ROC) curve analysis was performed to find an optimal cut-off value, sensitivity and specificity of PLR. For all the abovementioned tests, p < 0.05 was considered as statistically significant. An interobserver reliability analysis with the Cohen’s kappa statistic was performed to identify the consistency among the raters. 

## 3. Results

Eighty-seven patients diagnosed with COVID-19 (coronavirus disease 2019) through PCR were grouped by age, comprising 32 patients between 20 and 40 years old, 32 patients between 41 and 60 years old, and 23 patients over 61 years old. CT findings were detected in 56 patients with COVID-19 (64.4%) at the time of their admission, yet no evidence of COVID-19 involvement in thorax CTs was found for the remaining 31 patients (35.6%).

### 3.1. Distribution of lung findings and quantitative scoring

Multilobar involvement was observed in 45 of 56 (80.35%) patients with lung involvement in their CTs. The ground-glass densities detected in the CTs of 56 patients were characterized by a peripheral location only for 43 patients (76.78%) while 13 patients (23.12%) showed both central and peripheral locations. Crazy paving findings were identified for 15 patients (26.78%), consolidation for 11 patients (19.64%), halo findings for four patients (7.14%), and air bubble findings for one patient. Four patients (7.14%) had pleural effusion while 10 patients (17.85%) had septal thickening or pleural shrinkage at the lower-lobes lesion level. For eight patients (14.28%), a lymph node with a short axis size of over 1 cm was detected in the mediastinum.

COVID-19-positive patients’ lung involvement was scored for each lobe and lingula. Accordingly, the mean lung score was calculated as 6.63 ± 4.70 (1–23) by observer 1 and 6.55 ± 4.65 (1–23) by observer 2 ( *K* = 0.824–1.000). The interobserver reliability for lung parenchymal involvement was quite high. The mean score of the patients between 20 and 40 years old (n = 32) was 1.97 ± 3.93, whereas the mean score of the patients between 41 and 60 years old (n = 32) was 5.41 ± 5.85, and the mean score of patients over 61 years old (n = 23) was 5.87 ± 4.26. A statistical significance was found between increases in age and increases in scores for the lung findings among COVID-19 patients ( *p* = 0.003). Ground-glass densities and other findings indicating lung involvement were located more commonly in both the lower lobes (78.6%) and located less in the middle lobe (64.29%) and the lingula (51.78%) (Table 1).

**Table 1:** Quantitative scoring in COVID-19 pneumonia.

Right					Left				
		O1	O2	Kappa			O1	O2	Kappa
Superior lobe	No signs of pneumonia	17	17	0.824	Superior lobe	No signs of pneumonia	22	22	0.945
	0%–25%	22	21			0%–25%	24	23	
	26%–50%	11	12			26%–50%	6	7	
	51%–75%	5	5			51%–75%	3	3	
	76%–100%	1	1			76%–100%	1	1	
Middle lobe	No signs of pneumonia	20	24	0.882	Lingula	No signs of pneumonia	27	27	1.000
	0%–25%	29	25			0%–25%	22	22	
	26%–50%	5	5			26%–50%	6	6	
	51%–75%	1	1			51%–75%	0	0	
	76%–100%	1	1			76%–100%	1	1	
Inferior lobe	No signs of pneumonia	12	12	0.952	Inferior lobe	No signs of pneumonia	12	12	0.952
	0%–25%	11	12			0%–25%	16	17	
	26%–50%	20	20			26%–50%	17	18	
	51%–75%	12	11			51%–75%	11	9	
	76%–100%	1	1			76%–100%	0	0	

O1= Observer 1, O2= Observer 2.

### 3.2. CT findings and lab analysis

For patients with COVID-19 imaging findings in thorax CTs, a significant decrease was observed in white blood cell (WBCs), neutrophil, lymphocyte, and monocyte counts compared to patients without lung involvement (Table 2). To achieve homogenous distribution, the scoring of lung involvement was divided into two groups as 0–8 points (disease with mild involvement) and 9–24 points (disease with severe involvement). Within this framework, lymphocyte counts for patients with severe involvement were significantly lower than for patients with mild involvement ( *p* = 0.0001), just as PLR was found to be significantly lower for patients with severe involvement than patients with mild involvement ( *p* = 0.001). Further, a significant decrease was observed in lymphocyte counts ( *p* = 0.005) and platelet count ( *p* = 0.0001) for patients with crazy paving CT findings.

**Table 2:** Comparison of COVID-19 CT findings with hemogram data.

	CT findings				Scoring				Crazy paving				Consolidation			
	Positive(n=56)Mean±SD	Negative(n=31) Mean±SD	p	z	1–8(n=32) Mean±SD	9–23(n=24) Mean±SD	p	z	Positive(n=15) Mean±SD	Negative(n=41) Mean±SD	p	z	Positive (n=11) Mean±SD	Negative (n=45) Mean±SD	p	z
WBC(K/uL)	6.45±2.75	9.46±2.86	0.0001	–4.68	7.46±3.16	5.53±2.63	0.009	–2.597	5.15±2.08	7.45±3.15	0.002	–3.096	5.26±2.09	7.22±3.19	0.066	–1.839
Neutrophil(K/uL)	4.31±2.43	6.50±2.91	0.0001	–4.09	4.37±2.88	3.86±2.51	0.237	–1.182	3.49±1.93	4.39±2.89	0.042	–2.034	3.65±1.75	4.35±2.90	0.237	–1.181
Lymphocyte(K/uL)	1.59±0.85	2.24±1.02	0.0001	–3.492	1.97±1.01	1.25±0.47	0.0001	–3.709	1.22±0.44	1.84±0.99	0.005	–2.837	1.24±0.54	1.76±0.99	0.088	–1.705
Monocyte(K/uL)	0.45±0.19	0.54±0.22	0.77	–1.769	0.47±0.20	0.37±0.19	0.051	–1.948	0.35±0.18	0.46±0.20	0.016	–2.406	0.49±0.23	0.44±0.20	0.687	–0.402
Platelet(K/uL)	225.75±74.65	248.29±48.10	0.03	–2.172	223.00±54.60	207.00±93.26	0.158	–1.948	163.00±45.18	236.00±65.23	0.0001	–3.826	189.00±92.09	227.00±62.79	0.115	–1.577
NLR	3.51±3.04	4.37±5.13	0.873	–0.16	2.24±4.06	2.71±3.47	0.027	–2.213	2.62±2.42	2.36±4.15	0.286	–1.067	2.69±1.89	2.45±4.11	0.474	–0.715
PLR	167.85±78.65	140.84±90.23	0.006	–2.747	122.80±71.30	181.23±96.45	0.001	–3.267	129.54±81.24	131.02±84.37	0.406	–0.832	146.40±56.36	130.21±86.92	0.235	–1.188
MLR	0.34±0.22	0.30±0.21	0.178	–1.347	0.21±0.21	0.25±0.21	0.122	–1.548	0.24±0.18	0.22±0.21	0.633	–0.478	0.25±0.18	0.22±0.21	0.697	–0.390

N= Number of patients, WBC= White blood cell, NLR= Neutrophil-lymphocyte ratio, PLR= Platelet- lymphocyte ratio, MLR= Monocyte- lymphocyte ratio, CT = Computed tomography, COVID-19= Coronavirus disease, SD= Standart deviation.

### 3.3. Relationship between thymus and COVID-19 findings

Of 87 COVID-19-positive patients, 6 had thymus attenuation at grade 0, 18 at grade 1, 29 at grade 2, and 34 at grade 3.The interobserver reliability of grading thymus fat infiltration seemed remarkably high ( *K* : 0.991). As summarized in Table 3, a significant relationship was identified between the increased thymus fat component and the presence of COVID-19 lung involvement via CT ( *p* = 0.029). Another significant relationship was found between a decrease in the thymus attenuation degree and multilobar involvement ( *p* = 0.001). A moderate positive correlation was found between the degree of thymus fat infiltration and the presence of pulmonary findings ( *r* = 0.461), and a high positive correlation was found between the degree of thymus fat infiltration and age ( *r* = 0.603) ( *p* < 0.0001). A moderate negative correlation was found between the degree of thymus fat infiltration and the lymphocyte counts ( *r* = –0.461, *p* = 0.001), and a moderate positive correlation was found between the degree of thymus fat infiltration and NLR ( *r* = 0.489, *p* < 0.0001) (Table 4).

**Table 3:** Comparison of the degree of thymus fat involution with CT findings.

The degree of thymus fat involution	CT findings		Scoring		Ground glass densities		Multilobar involvement		Crazy paving		Consolidation	
	Negative (n=31)	Positive (n=56)	1–8(n=32)	9–23(n=24)	Peripheral (n=43)	Peripheral + central (n=13)	Negative (n=11)	Positive (n=45)	Negative (n=41)	Positive (n=15)	Negative (n=45)	Positive (n=11)
Grade 3	7	27	11	16	21	6	1	26	16	11	22	5
Grade 2	10	19	12	7	14	5	5	14	15	4	14	5
Grade 1	11	7	6	1	5	2	2	5	7	0	7	0
Grade 0	3	3	3	0	3	0	3	0	3	0	2	1
p	0.029		0.050		0.768		0.001		0.084		0.453	

N= Number of patients, CT = Computed tomography

**Table 4:** Correlation of blood cell count with the degree of attenuation of thymus tissue.

		WBC	Neutrophil	Lymphocyte	Monocyte	Platelet	NLR	PLR
The degree of attenuation of thymus tissue	r	–0.064	–0.176	–0.425	0.181	–0.304	0.489	0.314
	p	0.642	0.195	0.001	0.181	0.023	0.000	0.018

WBC= White blood cell, NLR= Neutrophil-lymphocyte ratio, PLR= Platelet-lymphocyte ratio.

### 3.4. The univariate logistic regresion models and ROC curve analysis

**Figure 3:**
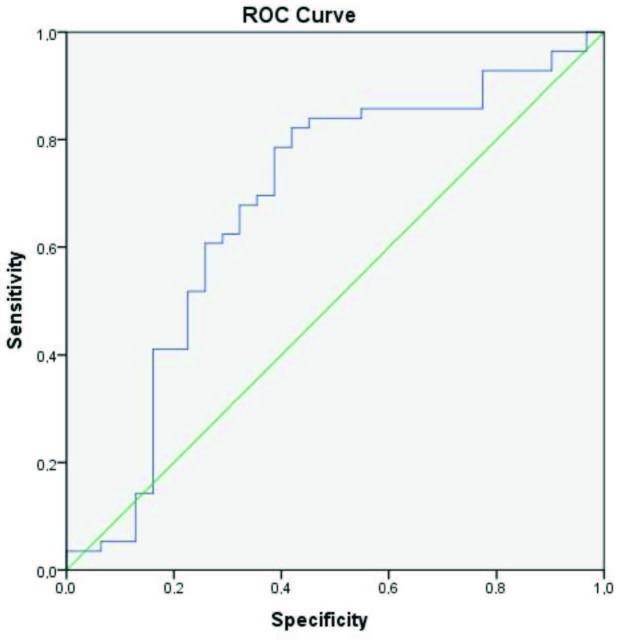
ROC curve for platelet to lymphocyte ratio.

Univariate analysis indicated that neutrophil ( *OR* = 0.740, 95% *CI* , 0.616–0.890, *p* < 0.0001), lymphocyte ( *OR* = 0.466, 95% *CI* , 0.271–0.802, *p* = 0.006), WBC ( *OR* = 0.702, 95% *CI* , 0.587–0.840, *p* = < 0.0001), and thymus fat involution ( *OR* = 0.511, 95% *CI* , 0.310–0.841, *p* = 0.008) values positively correlated with lung involvement (Table 5). The area under ROC curve (AUC) was 0.679 (95% confidence interval, *CI* : 0.552–0.805) for PLR. Figure 3 shows that the cut-off PLR value for lung involvement was 103.88 while sensitivity was 84% and specificity was 55% ( *p* = 0.006).

**Table 5:** Results of univariatelogistic regression for lung involvement.

	β	p value	Odds ratio	Confidence interval	
WBC	–0.354	0.0001	0.702	0.587	0.840
Neutrophil	–0.301	0.001	0.740	0.616	0.890
Lymphocyte	–0.763	0.006	0.466	0.271	0.802
Platelet	–0.005	0.140	0.995	0.988	1.002
Monocyte	–2.175	0.052	0.114	0.013	1.022
NLR	–0.054	0.336	0.947	0.848	1.058
PLR	0.004	0.155	1.004	0.998	1.011
MLR	0.982	0.383	2.670	0.294	24.284

WBC= White blood cell, NLR= Neutrophil-lymphocyte ratio, PLR= Platelet-lymphocyte ratio, MLR= Monocyte-lymphocyte ratio.

## 4. Discussion

Our study indicates that CT findings and quantitative CT scores tend to increase in parallel with increasing fat attenuation in the thymus. We also noted a significant relationship between increased fat attenuation in the thymus and decreased lymphocyte counts in the patients of our study group. The most important cause of fat infiltration in the thymus is age, and COVID-19 shows higher lung involvement for patients of older ages. In our study, we found a statistically significant positive correlation between age and thymus fat infiltration. In addition, our study found a moderate positive correlation between the degree of thymus fat infiltration and the presence of pulmonary findings. In this study, in 23 patients over the age of 40 years with mild lung involvement, thymus tissue attenuation was observed as grades 0 and 1. For three patients over the age of 40 years, their thymus tissue was almost completely solid, and lung involvement was not observed. We can, thus, conclude that thymus size and amounts of fat involution also play a key role in coronavirus disease 2019, in addition to amounts of T lymphocytes.

Neutrophil increases and decreases in lymphocyte counts, as well as multilobar involvement, were documented as more pronounced among elderly patients than young patients. Changes in the respiratory system’s anatomical and physiological functions, in addition to a weakened defense barrier, were claimed to be potentially responsible for the higher severity of COVID-19 among elderly patients [16,17]. Likewise, lung involvement scores were ultimately found to be higher among the elderly group in our study. Amounts of thymus fat involution were also higher among the elderly group compared to their younger counterparts, and decreased lymphocyte counts were more evident.

A higher amount of proinflammatory cytokines (TNF-alpha, IL-1, IL-6) and chemokines were noted in patients with severe COVID-19 findings compared to patients with mild symptoms [18]. In COVID-19 pathogenesis, lung findings have been reported to depend on a rise in serum cytokine and chemokine levels. In addition, a decreasing lymphocyte count and an increasing neutrophil-lymphocyte ratio were observed for patients with severe symptoms [19]. Some studies also established NLR as an independent risk factor for mortality and prognosis, notably in men [10]. No significant change occurred in lymphopenia B cells and CD8 + Tlenfocyte counts for severe COVID-19 patients, while a decrease in CD4 + T lymphocyte counts was noted [20]. Some research predicted that lymphopenia could be caused by direct virus attacks, attacks by inflammatory mediators, or lymphocytes’ passage from peripheral blood to the lungs [21,22]. In our study, we established a significant decrease in lymphocyte counts for patients with COVID-19 whose imaging findings were positive, whose CT findings’ severity increased, and whose crazy paving findings were detected in CTs. On the other hand, we did not find any significant NLR rate differences in CT findings and quantitative scoring. We suggest that the thymus plays a critical role in this disease in light of the data, such as lymphopenia and NLR, utilized for COVID-19 patients’ prognoses. Qin et al. documented that CD4 + T cells tend to decrease without any significant changes in B lymphocyte and CD8 + T lymphocyte counts [19].

The platelet-lymphocyte ratio (PLR) is one marker of systemic inflammation. Qu et al. suggested that the length of a patient’s hospital stay positively correlated with their age and platelet levels, though negatively correlating with lymphocyte levels. In their study, which calculated a PLR cut-off value at PLR > 126.7, COVID-19 patients’ high PLR was reported to extend the length of their hospital stays, and PLR was associated with disease prognosis [11]. In our study, meanwhile, we found 84% sensitivity and 55% specificity, while our PLR cut-off value was 103.88 for lung involvement ( *p* = 0.006). PLR proved to be significantly higher for patients with severe disease involvement than patients with mild disease involvement. Given the increased CT findings, we linked these patients’ PLR increases to lymphopenia, and we observed a significant decrease in platelet counts for patients with crazy paving findings via CT.

In our study, we quantitatively scored COVID-19 thorax CT findings by calculating each lung lobe’s proportion of involvement. As a result, we found mean scores for involvement of 6.63 ± 4.70 by observer 1 and of 6.55 ± 4.65 (1–23) by observer 2. Within the context of our study group, a significant increase was observed in lung findings scores, with increases according to age. Previous research that had based quantitative evaluation on a scoring system ultimately revealed the prognosis of patients with high scores to be poor [6,14,23].

This study was planned using an interobserver design in which CT images of COVID-19 patients were assessed by two radiologists. Excellent agreement was achieved between the two interobservers for quantitative scoring of the CT findings, patients’ thymus imaging features, and the CT findings themselves.

Our study involved some limitations. The first limitation concerned the study’s retrospective characteristic, whereas the second limitation was our inability to follow up on our COVID-19 patients’ CT findings. CT examinations were not repeated-not only because these patients’ follow-up imaging findings abound in the literature in detail but also to prevent patients’ re-exposure to the radiation of a CT scan. Third, these patients’ follow-up lab data were not evaluated; instead, patients’ CTs at the time of their admission to the emergency department were compared with their first lab data. Moreover, patients’ thymic volumes could not be measured. While our COVID-19 patients’ general symptoms were characterized by shortness of breath and coughing, an optimal quantitative evaluation could not be performed due to breathing-related artifacts during CT scans. Another limitation was that patients in the study group were not grouped according to their age and correlations in each age group were not calculated separately. A separate correlation calculations for each age could be helpful in demonstrating the relationship between COVID-19 and thymus fat involution, regardless of age. Finally, the type and duration of treatment for these patients, as well as the data’s effect on the duration of their hospitalization upon their admission to the emergency department, were not evaluated.

In conclusion, our study shows that imaging findings’ severity correlates significantly with COVID-19 patients’ platelet-lymphocyte ratios and that decreased platelet and lymphocyte counts lead to observable increases in imaging findings. The severity of imaging findings for COVID-19 patients significantly correlates with the level of fat involution in patients’ thymus tissue. Future studies conducted on the thymus would contribute substantially to coronavirus disease 2019 treatment and prognosis.
